# Ethical guidelines for publishing in the *Journal of Cachexia, Sarcopenia and Muscle*: update 2019

**DOI:** 10.1002/jcsm.12501

**Published:** 2019-10-29

**Authors:** Stephan von Haehling, John E. Morley, Andrew J. S. Coats, Stefan D. Anker

**Affiliations:** ^1^ Department of Cardiology and Pneumology University of Göttingen Medical Center Göttingen Germany; ^2^ Divisions of Geriatric Medicine and Endocrinology Saint Louis University School of Medicine St. Louis MO USA; ^3^ San Raffaele Pisana Scientific Institute Rome Italy; ^4^ Department of Cardiology (CVK) and Berlin Institute of Health Center for Regenerative Therapies (BCRT), German Centre for Cardiovascular Research (DZHK) partner site Berlin Charité Universitätsmedizin Berlin Germany

**Keywords:** Ethical guidelines, Publishing

## Abstract

This article details an updated version of the principles of ethical authorship and publishing in the *Journal of Cachexia, Sarcopenia and Muscle* (*JCSM*) and its two daughter journals *JCSM Rapid Communication* and *JCSM Clinical Reports*. We request of all author sending to the journal a paper for consideration that at the time of submission to *JCSM*, the corresponding author, on behalf of all co‐authors, needs to certify adherence to these principles. The principles are as follows:

- all authors listed on a manuscript considered for publication have approved its submission and (if accepted) approve publication in *JCSM* as provided;
- each named author has made a material and independent contribution to the work submitted for publication;
- no person who has a right to be recognized as author has been omitted from the list of authors on the submitted manuscript;
- the submitted work is original and is neither under consideration elsewhere nor that it has been published previously in whole or in part other than in abstract form;
- all authors certify that the submitted work is original and does not contain excessive overlap with prior or contemporaneous publication elsewhere, and where the publication reports on cohorts, trials, or data that have been reported on before the facts need to be acknowledged and these other publications must be referenced;
- all original research work has been approved by the relevant bodies such as institutional review boards or ethics committees;
- all relevant conflicts of interest, financial or otherwise, that may affect the authors' ability to present data objectively, and relevant sources of funding of the research in question have been duly declared in the manuscript;
- the manuscript in its published form will be maintained on the servers of *JCSM* as a valid publication only as long as all statements in the guidelines on ethical publishing remain true.
- If any of the aforementioned statements ceases to be true, the authors have a duty to notify as soon as possible the Editors of *JCSM*, *JCSM Rapid Communication*, and *JCSM Clinical Reports*, respectively, so that the available information regarding the published article can be updated and/or the manuscript can be withdrawn.

The *Journal of Cachexia, Sarcopenia and Muscle* (*JCSM*) is a peer‐reviewed international journal dedicated to publishing materials that are related to cachexia and sarcopenia as well as to body composition and its physiological and pathophysiological changes during the life span and in different illnesses from all fields of the life sciences. *JCSM* was founded in 2010 and has attracted interest by both clinicians and researchers, which is buttressed by increasing download numbers, citations, and submissions and by an increasing impact factor.^1^, ^2^ Submissions to *JCSM* are now coming from and interest in the field of body wasting is embracing the entire globe.^3^, ^4^, ^5^

The authors of these ethical guidelines, who are editing five peer‐reviewed journals including—besides *JCSM*—the *Journal of the American Medical Directors Association*, *ESC Heart Failure*, and the two new daughter journals *JCSM Clinical Reports* and *JCSM Rapid Communications*, share the view that academic publishing depends, to a great extent, on trust between authors, reviewers, editors, and the academic and lay public.^6^, ^7^, ^8^ Scientific publications are made in an environment of many competing interests that may be commercial (in many different ways), intellectual, and/or political in nature. Some of these competing interests create conflicts of interest. In this context, the Editors of *JCSM* see it as their duty to promote transparency and research integrity. And, as this is an ongoing effort, we wish to recall the validity and update the statements on ethical publishing made by us before.^9^, ^10^, ^11^

All authors wishing to publish in *JCSM* and its two daughter journals *JCSM Clinical Reports* and *JCSM Rapid Communications* are required to certify that they comply with the guidelines of publishing in *JCSM* outlined in the succeeding text. This should be performed by stating as part of the published manuscript that ‘All authors of this manuscript comply with the guidelines of ethical authorship and publishing in the Journal of Cachexia, Sarcopenia and Muscle’, providing reference to these guidelines by referencing this article. Specifically, the corresponding author, on behalf of all co‐authors,

- certifies that all authors listed on a manuscript considered for publication have approved its submission and (if accepted) approve publication in *JCSM* as provided;
- certifies that each named author has made a material and independent contribution to the work submitted for publication—authorship credit should be based only on, first, a substantial contribution to conception and design, or acquisition of data, or analysis and interpretation of data, and second, drafting the article or revising it critically for important intellectual content;
- certifies that no person who has a right to be recognized as author has been omitted from the list of authors on the submitted manuscript;
- certifies that the submitted work is original and is neither under consideration elsewhere nor that it has been published previously in whole or in part other than in abstract form;
- certifies that all authors certify that the submitted work is original and does not contain excessive overlap with prior or contemporaneous publication elsewhere, and where the publication reports on cohorts, trials, or data that have been reported on before the facts need to be acknowledged and these other publications must be referenced (of note, the degree of overlap and difference must be fully described and justified; excessive overlap that is not justified in a cover letter will lead to rejection or withdrawal of the manuscript);
- certifies that all original research work has been approved by the relevant bodies such as institutional review boards or ethics committees (of note, this rule is not applicable for review articles or editorial comments that give reference to other people's work);
- certifies that all relevant conflicts of interest, financial or otherwise, that may affect the authors' ability to present data objectively, and relevant sources of funding of the research in question have been duly declared in the manuscript;
- certifies that the manuscript in its published form will be maintained on the servers of *JCSM* as a valid publication only as long as all statements in the guidelines on ethical publishing remain true.
- If any of the aforementioned statements ceases to be true, the authors have a duty to notify as soon as possible the Editors of *JCSM*, *JCSM Rapid Communication*, and *JCSM Clinical Reports*, respectively, so that the available information regarding the published article can be updated and/or the manuscript can be withdrawn.

We will continue our editorial work for the *JCSM* journal family, and the increasing number of submissions underscores the continuing need for such a niche journal and its two daughter journals, *JCSM Rapid Communication* and *JCSM Clinical Reports*.^12^ This is particularly so, because diagnostic tools have improved and overall awareness and research in cachexia and sarcopenia have increased. Nevertheless, there still remains a great unmet medical need, because cachexia and sarcopenia are still associated with an unacceptably high burden of morbidity and mortality affecting patients, families, caregivers, and healthcare systems and no new therapies exist yet.

Despite the existing wish to advance our knowledge, we recognize that the world is not free of scientific misconduct, which can come in many ways. We are happy to report that only very few problems have arisen in the past seven years, and no published paper had to be withdrawn so far. The Editors of *JCSM* aim to continue to keep a close eye on these issues, and we need reviewers to help in this. We simply ask that authors play fair to the academic community and the public at large and then none of this will ever be an issue. We want to take a lead in identifying, notifying, investigating, and reporting all occurrences of scientific misconduct related to *JCSM*. We will withdraw papers, if significant problems come to our attention. This shall be no surprise to anyone when it has to occur. Hence, we request that these ethical guidelines for publishing in *JCSM* and its two daughter journals are cited in each article. As stated before, academic publishing is built on trust. We want *JCSM* to be a trusted and valued source of best available scientific knowledge in the field. We hope that, together, we can achieve this objective and our journal can succeed in this ambition.
